# Glucocorticoid-dependent multiple sclerosis overlapping anti-NMDA receptor encephalitis: a case report and literature review update

**DOI:** 10.1007/s10072-023-07034-x

**Published:** 2023-09-18

**Authors:** Bo Yang, Nengwei Yu

**Affiliations:** 1 Department of Center for Psychosomatic Medicine, Sichuan Provincial Center for Mental Health, Sichuan Provincial People’s Hospital, University of Electronic Science and Technology of China, Chengdu, China; 2 Department of Neurology, Sichuan Provincial People’s Hospital, University of Electronic Science and Technology of China, No. 32 West Second Section of First Loop, Qingyang District, Chengdu City, Sichuan Province China

**Keywords:** Anti-NMDA receptor encephalitis, Case report, Glucocorticoid-dependent, Multiple sclerosis

## Abstract

**Background:**

Previous studies suggest a relationship between central nervous system inflammatory demyelinating diseases and anti-N-methyl-d-aspartate receptor (NMDAR) encephalitis. Also, the overlap between anti-NMDAR encephalitis and multiple sclerosis (MS) has been reported. However, the pathogenesis and clinical characteristics are still obscure.

**Case presentation:**

A 33-year-old woman presented with diplopia and sensory ataxia at the onset. The cerebrospinal fluid (CSF) anti-NMDAR antibodies were positive (1:3.2), and nuclear magnetic resonance imaging (MRI) showed bilateral centrum ovale and lateral ventricle demyelinating lesions. Therefore, she was diagnosed with anti-NMDAR encephalitis. After administering intravenous immunoglobulin and oral prednisone, her lesions disappeared, and symptoms were relieved. The condition was maintained with a low dose of prednisone, but her lesions reappeared on MRI. Consequently, immunomodulatory therapy of mycophenolate mofetil was initiated. However, she developed dysarthria and right limb ataxia after 10 months with a positive CSF anti-NMDAR antibody (1:1) and positive oligoclonal band. The MRI showed symmetrical multiple demyelinating lesions. Considering the MS diagnosis, her neurological dysfunction again improved significantly after intravenous methylprednisolone. Unfortunately, her symptoms aggravated for the second time when teriflunomide was started. Finally, her condition was controlled again with oral prednisone.

**Conclusions:**

Consistent with previous cases of overlapping anti-NMDAR encephalitis and MS, patients often show atypical symptoms on MRIs and immunological tests. The overlap cannot be arbitrarily treated because of the recurrence of previous diseases. Long-term follow-up, dynamic antibody monitoring, and MRI examination are crucial for these patients. The special dependency of the patient on glucocorticoids in this study has been rarely reported, which may guide the treatment of insensitivity to disease-modifying therapy in recurrent overlapping anti-NMDAR encephalitis and MS.

## Background

Multiple sclerosis (MS) is an autoimmune inflammation-mediated demyelinating disease of the central nervous system (CNS). The incidence rate of MS has increased in recent years and has become an essential cause of nontraumatic disability in young people [1]. Dissemination in space and time (DIT) is a significant clinical feature of MS, where lesions often involve the optic nerve, brainstem, cerebellum, cerebral hemisphere, and spinal cord [2]. Generally, diagnosing MS is not difficult. However, a long-term clinical follow-up may be required for atypical cases with first onset to determine the final diagnosis.

Anti-NMDAR encephalitis is the most common diagnosis of autoimmune encephalitis. The main clinical manifestations of anti-NMDAR encephalitis include mental and behavioral disorders, cognitive dysfunction, epilepsy, dyskinesia, and autonomic dysfunction [3]. Previous studies have reported the overlaps between anti-NMDAR encephalitis and central demyelinating diseases such as neuromyelitis optica spectrum disorder, acute disseminated encephalomyelitis, optic neuritis, and MS. However, these reports are still rare and have unclear clinical features [4]. Therefore, this study aimed to report a case of overlapping anti-NMDAR encephalitis and MS. We also comprehensively gathered similar reports to analyze the clinical characteristics of overlapping anti-NMDAR encephalitis and MS.

## Case presentation

### First admission (August 2021)

A 33-year-old woman developed a transient fever 1 month after vaccination for the 2019 novel coronavirus disease (COVID-19), with a maximum temperature of 38.5 °C. She later developed diplopia and unstable gait, successively, and was admitted to our hospital on August 30, 2021. The neurological examination showed limitation of the left inferior movement of the left eyeball, a four grade of right lower limb muscle strength, and Romberg sign. Abnormalities were not detected on hematology and serum biochemistry, including infection, immunity, endocrine system, and tumor. Abdominal and pelvic ultrasound, electrocardiogram, and chest X-ray also showed no abnormalities. Brainstem auditory-evoked potential, visual-evoked potential, electroneurography, and electroencephalogram were normal. Her Hess screen examination suggested superior oblique muscle paralysis of the left eye. The fundus photography showed the left eyeball external rotation. A somatosensory evoked potential indicated that the N22-P40 interval in the right lower limbs was prolonged.

**Fig. 1:**
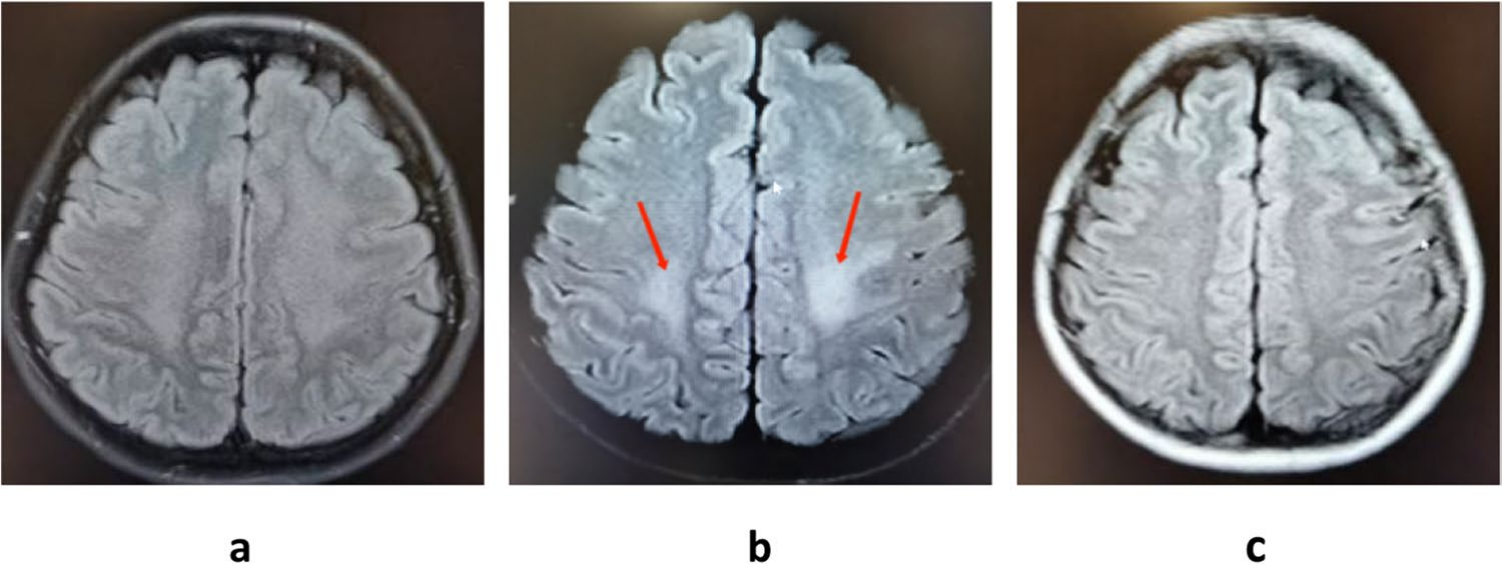
**a**Normal MRI on September 3, 2021;**b**demyelinating lesions in the centrum ovale majus and bilateral ventricle on September 30, 2021;**c**lesions dissipation after IVIG and prednisone on November 1, 2021

On September 3, 2021, magnetic resonance imaging (MRI) of the head, entire spinal cord, and brainstem was normal (Fig.1a). A cerebrospinal fluid (CSF) examination showed a protein level of 1157.61 mg/L and 46 × 10^6^/L total nucleated cells with 97% lymphocytes. The serum and CSF central nervous demyelinating antibody, peripheral nerve ganglioside antibody, oligoclonal band (OCB), immunoglobulin G (IgG) index, and intrathecal synthesis index were all found to be within normal ranges. We considered a diagnosis of viral brainstem encephalitis, and she was treated with intravenous ganciclovir 0.25 g every 12 h. However, her symptoms did not improve after 10 days of treatment. On September 14, 2021, a CSF recheck showed 35 × 10^6^/L total nucleated cells with 97% lymphocytes and a protein level of 963.47 mg/L. The CSF metagenomic next-generation sequencing (mNGS) did not show a pathogenic microbial gene sequence. Later, unfortunately, the patient’s right lower limb muscle strength progressively decreased.

On September 26, 2021, the reexamination of the CSF suggested a nucleated cell count of 11 × 10^6^/L with 90% lymphocytes and a protein level of 892 mg/L. At this point, further autoimmune encephalitis antibody detection suggested that anti-NMDAR antibody IgG in CSF was positive with a titer of 1:3.2, and the serum and CSF paratumor antibodies, anti-myelin oligodendrocyte glycoprotein (MOG) autoantibodies, were negative. Furthermore, on September 30, 2021, the patient’s head MRI showed abnormal signals in the centrum ovale majus and bilateral ventricle, indicating demyelinating lesions (Fig.1b). After considering a diagnosis of anti-NMDAR encephalitis, the patient received intravenous immunoglobulin (IVIG) (0.4 g/kg ⋅ d) for 5 days and oral prednisone 50 mg/d. After 1 month, her lower limb weakness and walking instability disappeared, and only diplopia partly remained. In addition, previous abnormal signals on MRI were completely absorbed (Fig.1c; on November 1, 2021). As a result, the dose of prednisone was reduced regularly.

### Second admission (April 2022)

**Fig. 2:**
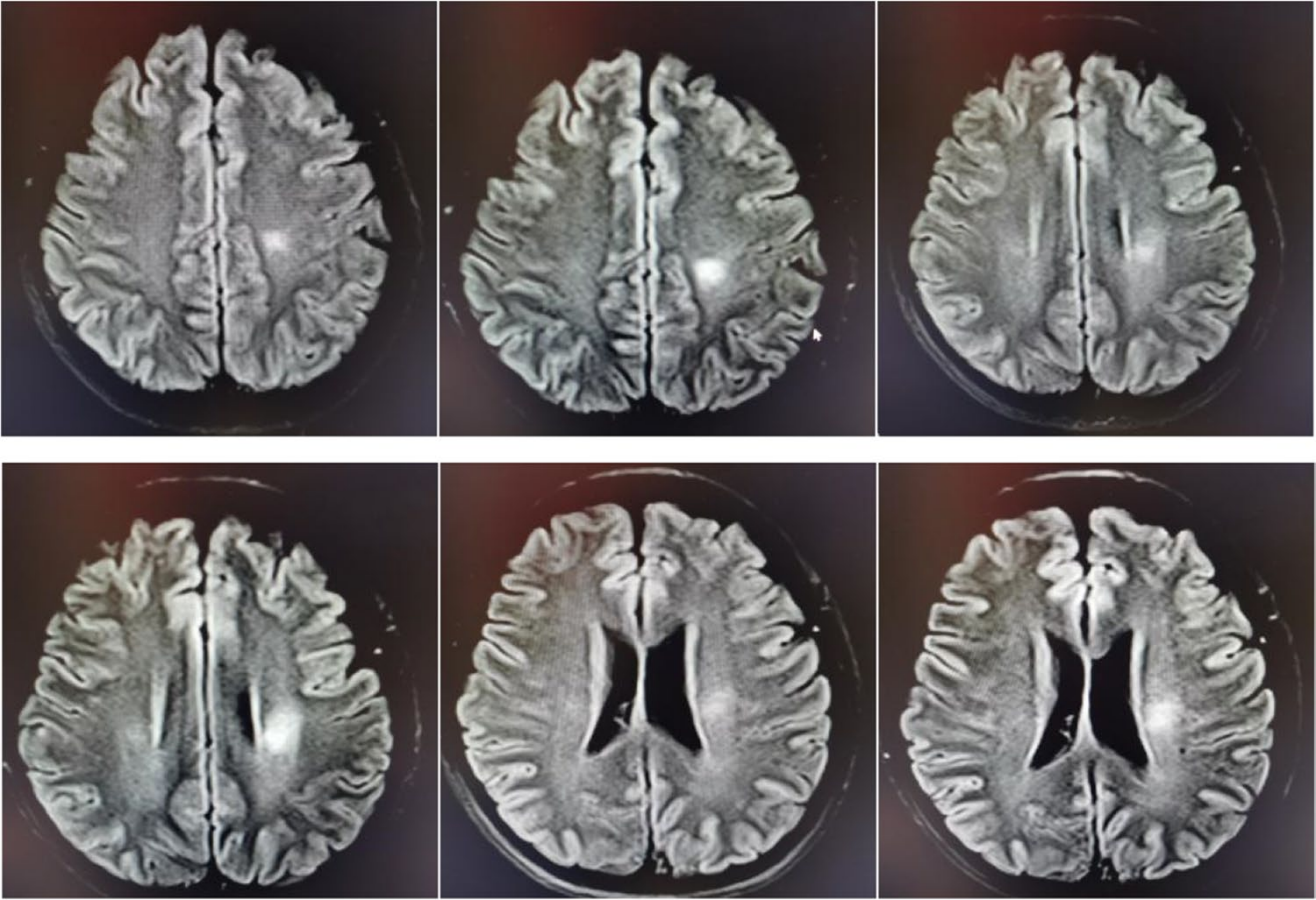
The MRI manifested abnormal signals in the left lateral ventricle and centrum semiovale on April 10, 2022

During the course of prednisone dose reduction in this patient, her diplopia was never completely relieved. When the prednisone dose was reduced to 15 mg/d, her walking instability recurred. The patient was readmitted, and a CSF follow-up examination showed normal nucleated cells and proteins. The titer of CSF anti-NMDAR antibody IgG was 1:1. At the same time, we examined the anti-MOG autoantibodies in CSF and serum, which were negative. In addition, the MRI manifested abnormal signals in the left lateral ventricle and centrum semiovale, and the lesions were diffusion limited without enhancement (Fig.2). She then received mycophenolate mofetil immunotherapy 1 g/d, and prednisone dose was gradually reduced to withdrawal.

### Third admission (August 2022)

**Fig. 3:**
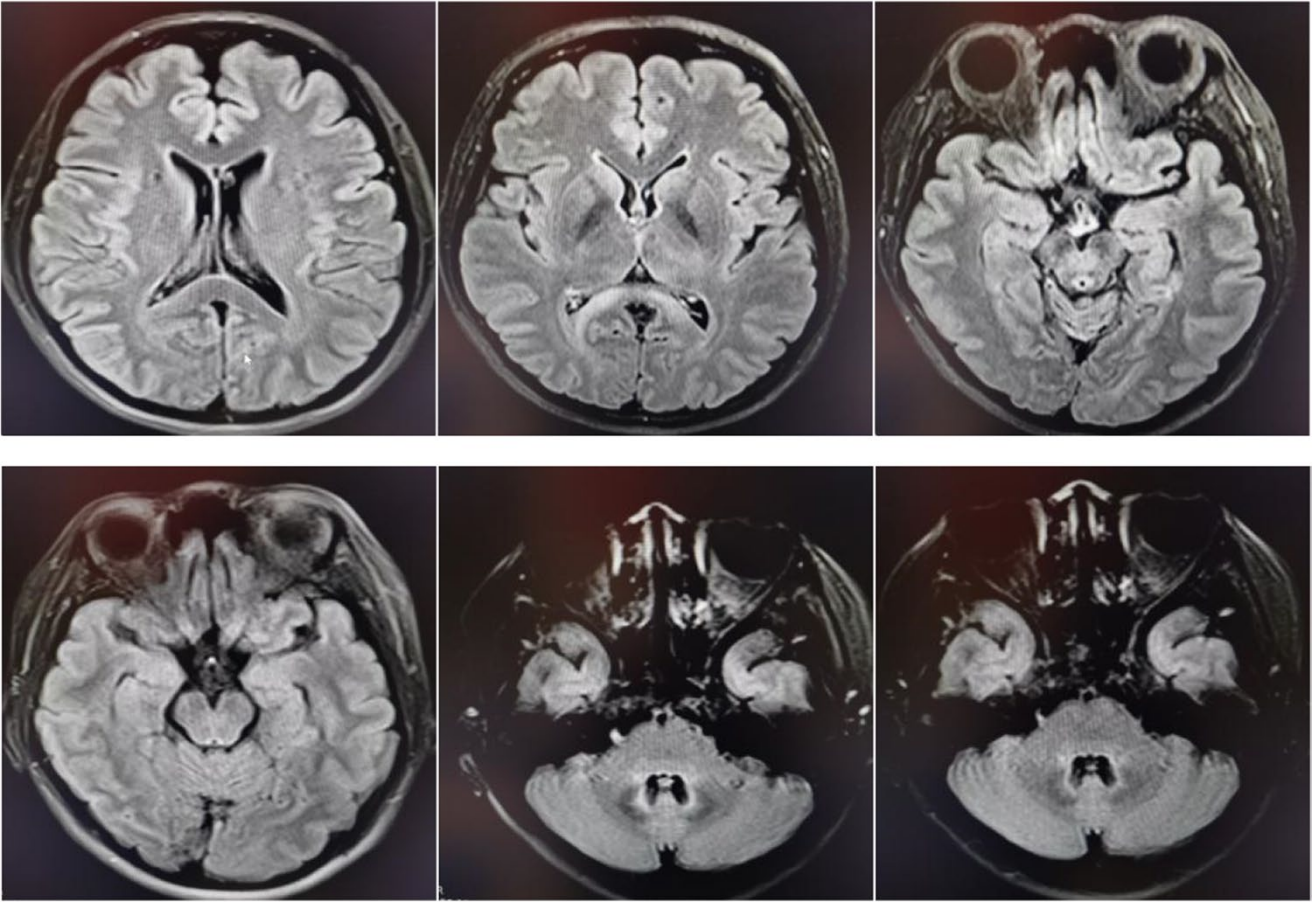
Multiple MRI lesions including enlarged left lateral ventricle and centrum semiovale lesions, symmetrical new lesions at the fourth ventricle, midbrain aqueduct, splenium of corpus callosum, and posterior horn of lateral ventricle

**Fig. 4:**
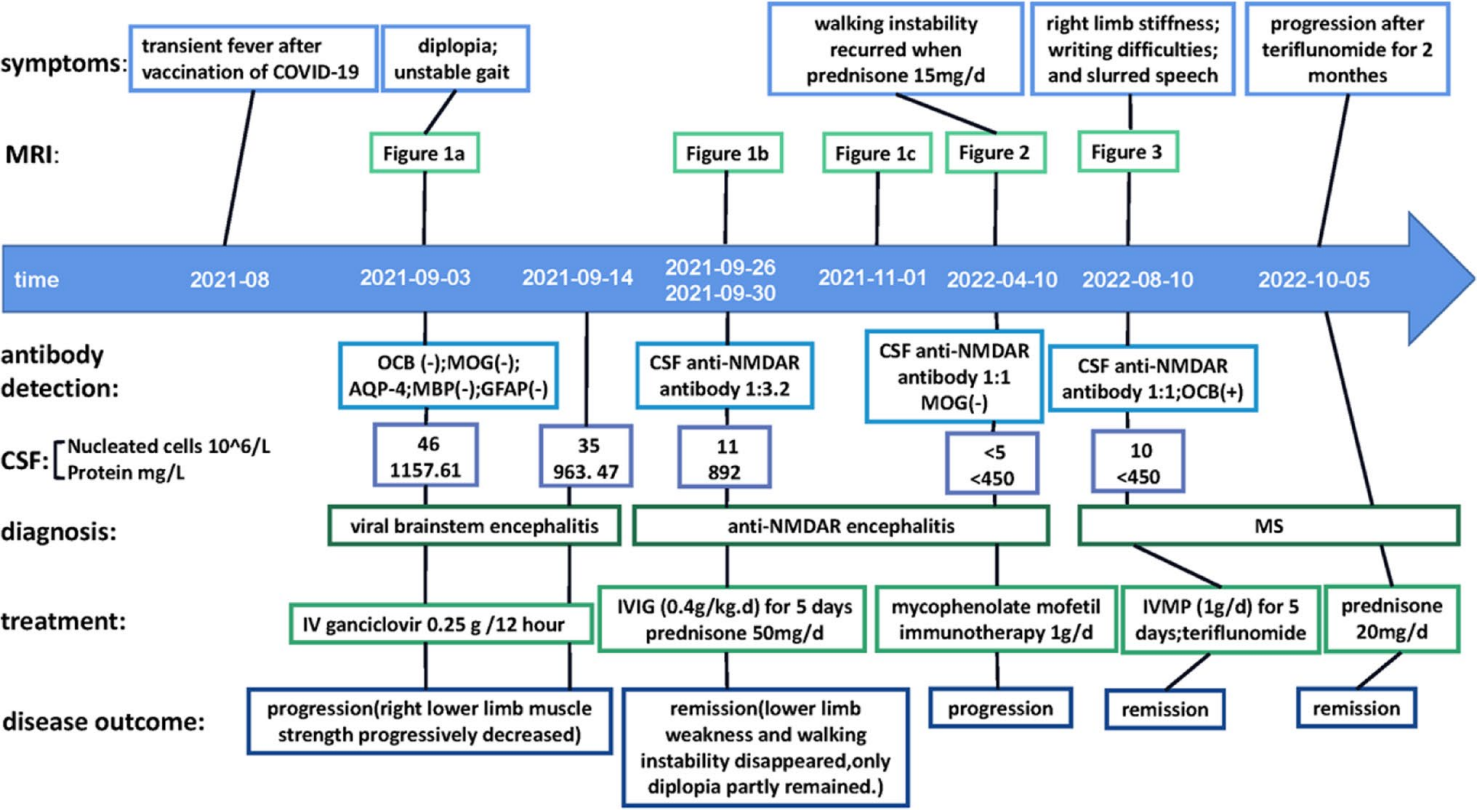
The course of this patient (CSF, cerebrospinal fluid; COVID-19, 2019 novel coronavirus disease; IVMP, intravenous methylprednisolone; IVIG, intravenous immunoglobulin; NMDAR, methyl-d-aspartate receptor; MS, multiple sclerosis)

Mycophenolate mofetil was maintained in the patient, but she developed a series of new symptoms, including right limb stiffness, writing difficulties, and slurred speech after only 1 month of prednisone withdrawal. The neurological examination at readmission suggested dysarthria and right limb ataxia. The number of nucleated cells was 10 × 10^6^/L with 90% lymphocytes in CSF, and anti-NMDAR antibody IgG was weakly positive (1:1). Oddly, we found positive CSF OCBs (10 bands) at this time. According to multiple MRI lesions, including enlarged left lateral ventricle and centrum semiovale lesions, symmetrical new lesions were found at the fourth ventricle, midbrain aqueduct, splenium of corpus callosum, and posterior horn of the lateral ventricle (Fig.3). We considered a diagnosis of MS. Consequently, the patient was given intravenous methylprednisolone (IVMP) (1 g/d) for 5 days, and her symptoms rapidly improved (Fig.4).

### Follow-up

Later, the patient was put on immune modification therapy with teriflunomide. However, her previous neurologic impairment symptoms were again exacerbated. Based on the repeated deterioration of this patient’s condition during the reduction and discontinuation of prednisone or methylprednisolone, glucocorticoid dependence was speculated, and prednisone was given again. The patient’s symptoms again improved. She is still receiving sustained prednisone therapy with a minimum effective dose of 20 mg/d.

## Literature review and discussion

We searched databases, such as PubMed, Embase, Sinomed, CNKI, and Wanfang, for case reports on MS and anti-NMDAR encephalitis published before August 2023. The search terms were “anti-N-methyl-D-aspartate receptor encephalitis” and “multiple sclerosis.” We found 18 related cases, and the clinical details are summarized in Table1. All patients were diagnosed with MS or anti-NMDAR encephalitis, successively, with an interval of 3 weeks to 15 years. It seemed that the diagnoses of many cases were converted from MS into anti-NMDAR encephalitis.

**Table 1:** Epidemiological, clinical，immunological, and imaging datas of previous cases of anti-NMDAR encephalitis overlapping MS

Case	Year	Age/Gender	Initial diagnosis	Initial symptoms	MRI findings	CSF or Serum anti-NMDAR antibody/OCB	Treatment	Prognosis	Later diagnosis	Later symptoms	MRI findings	CSF or Serum NMDAR antibody/OB	Duration between the two diagnosis	Treatment	Prognosis	References
1	2010	32/F	NMDAR	Seizure, unconsciousness	Normal	/	IVMP	Recovery	MS	Hypopsia, paresthesia	Multiple demyelination of MS	Serum anti-NMDAR antibody(+)	11 y	/	Recovery	[23]
2	2012	33/F	MS	Hypopsia	Multiple demyelination of MS	OCB+	IVMP	Relapsing-remitting	NMDAR	Seizure psychosis	Temporal lobe	CSF anti-NMDAR antibody(+)	3 y	IVMP, oral prednisolone	Recovery (6 m)	[24]
3	2014	33/M	MS	Diplopia, hemiparesis	Multiple demyelination of MS	OCB+	IVMP	Relapsing-remitting	NMDAR	Spasms., paresthesia	/	Serum anti-NMDAR antibody(+)	3 w	IVMP, rituximab	Recovery (12m)	[25]
4	2015	33/F	MS	Paresthesia, hypopsia ataxia	Multiple demyelination of MS	OCB+	Glatiramer acetate	Relapsing-remitting	NMDAR	Cognition, ataxia dysarthria	Brain atrophy brainstem cervical spinal cord	Serum anti-NMDAR antibody(+)	9 y	IVMP, azathioprine, cyclophosphamid, natalizumab, plasma exchange	Death (10 y)	[26]
5	2016	57/F	MS	Seizure, cognition	Multiple demyelination of MS	OCB+	IVIG IVMP	Recovery	NMDAR	Seizure, cognition	Bilateral frontal lobe, corpus callosum, left insula	CSF anti-NMDAR antibody(+)	1 y	IVMP, oral prednisolone	Recovery(3 m)	[27]
6	2017	32/F	NMDAR	Seizure, psychosis	Cerebral hemisphere, spinal cord	Serum anti-NMDAR antibody(+)	/	Partial improvement	MS	Diplopia prosopoplegia, ataxia	Multiple demyelination of MS	/	16 m	/	Recovery(10 m)	[28]
7	2017	29/M	NMDAR	Seizure, psychosis	Cerebral hemisphere	CSF/serum anti-NMDAR antibody(+)	NO	Recovery	MS	Lhermitte	Spinal cord	OCB(+)	2 y	/	/	[29]
8	2018	41/F	MS	Psychosis	/	OCB+	Dimethyl fumarate	Relapsing-remitting	NMDAR	Psychosis	\	Serum anti-NMDAR antibody(+)	7 y	IVMP, IVIg, rituximab	Memory impairment	[30]
9	2020	26/F	MS	Hemiplegia	Multiple demyelination of MS	OCB+	Interferone, fingolimod, natalizumab, teriflunomide	Recovery	NMDAR	Seizure, unconsciousness, psychosis	Ventricle	Serum anti-NMDAR antibody(+)	11 y	IVMP, plasma exchange, IVIg, rituximab	Recovery(12 m)	[31]
10	2020	19/F	MS	Hypopsia, Hemiplegia, Lhermitte	Multiple demyelination of MS	OCB+	IVIG, IVMP, interferon	Relapsing-remitting	NMDAR	Seizure, psychosis	Juxtacortical ventricle brainstem	OCB(+)\CSF and Serum anti-NMDAR antibody(+)	8 y	IVIG, IVMP, prednisolone	Recovery(5 m)	[32]
11	2020	33/F	NMDAR	Seizure, Psychosis	Normal	/	IVIG, IVMP	Recovery	MS	Hemiplegia	Multiple demyelination of MS	CSF anti-NMDAR antibody(+)	15 y	IVMP	Recovery	[33]
12	2020	33/F	MS	/	/	/	/	/	NMDAR	Psychosis	Cerebral hemisphere	CSF anti-NMDAR antibody(+)	3 y	IVIG, plasma exchange, prednisolone, monoclonal antibody	Recovery(/)	[34]
13	2021	34/M	MS	Paresthesia	Multiple demyelination of MS	OCB+	Dimethyl fumarate	Recovery	NMDAR	Tremor spasm	Temporal	OCB(+)\CSF anti-NMDAR antibody(+)	3 y	IVMP, rituximab	Recovery	[35]
14	2021	45/M	MS	/	/	/		Relapsing-remitting	NMDAR	Seizure, disorientation, fever	Temporal splenium ventricle	serum and CSF anti-NMDAR antibody(+)	7 y	IVIG, plasma exchange, rituximab	Cognitive impairment (21 m)	[36]
16	2021	16/F	NMDAR	Unconsciousness, Cognition	Multiple brain white matter	CSF anti-NMDAR antibody(+)	IVIG, IVMP	Relapsing-remitting	MS	Hemiplegia, diplopia, cognition	Multiple demyelination of MS	OCB(+)	2 y	Mycophenolate mofetil, IVMP, teriflunomide	Recovery (18 m)	[37]
17	2022	50/F	MS	Ataxia	Multiple demyelination of MS	/	Teriflunomide, rituximab	Relapsing-remitting	NMDAR	Ataxic paraplegia	Ventricle, juxtacortical, cortical areas	Serum anti-NMDAR antibody(+)	7 y	IVMP, oral prednisolone	Recovery (1 m)	[38]
18	2023	30/M	NMDAR	Psychosis	Normal	CSF/serum anti-NMDAR antibody(+)	IVMP	Recovery	MS	Diplopia, nystagmus vertigo	Semioval center ventricle	CSF anti-NMDAR antibody(+)/OCB(+)	8 m	IVMP, prednisone, dmycophenolate mofetil	Recovery (4 m)	[39]

*NMDAR*methyl-d-aspartate receptor,*MS*multiple sclerosis,*OCB*oligoclonal band,*IVMP*intravenous methylprednisolone,*IVIG*intravenous immunoglobulin

Vaccination and viral infection may be the inducing factors for anti-NMDAR encephalitis and MS [1, 5]. The patient in this study got a fever after receiving the COVID-19 vaccine. However, her diplopia and unstable gait did not conform to the characteristics of anti-NMDAR encephalitis. Consequently, we did not initially detect NMDAR antibodies. Her nervous system on MRI could not preclude NMDAR encephalitis. About 50% of patients with anti-NMDAR encephalitis have no abnormal signal on MRI [6]. Some studies suggested that CSF protein concentration and nucleated cell count might be normal or slightly elevated in MS and that a CSF protein level of more than 100 mg/dL or nucleated cell count of more than 50/mm^3^might indicate other diseases [7]. The first CSF examination of the patient in this study and negative OCBs did not support an MS diagnosis.

After antiviral therapy, a mild decrease in the patient’s protein level and nucleated cell count in CSF did not indicate viral meningoencephalitis sensitivity to antiviral therapy. In addition, her CSF mNGS, which had good diagnostic value for CNS infection, was also normal [8]. We later found that the CSF anti-NMDAR antibody was slightly positive (1:3.2). However, the patient’s MRI did not support anti-NMDAR encephalitis but supported MS. The MRIs of NMDA usually show high T2 and FLAIR signals in the hippocampus, cerebellum, cerebral cortex, frontal lobe, insula, and brainstem [5]. Unfortunately, we did not reinspect OCB due to previous negative results, but we later speculated that it might be positive at that time. We tentatively used IVIG and oral prednisone due to the patient’s refusal to IVMP, with effective results.

The inhibition of glucocorticoids for MS relapse may be transient [9,10]. Studies have suggested that IVMP can rapidly reduce the enhanced lesions of relapsing MS on MRI by about 96%. This benefit, however, may last for only 1 month and be followed by new or enlarged lesions [11]. As for anti-NMDAR encephalitis, delayed immunotherapy and high antibody titer may be the recurrent factors for anti-NMDAR encephalitis, and glucocorticoids can inhibit its recurrence [12,13]. The recurrent MRI lesions of the left lateral ventricle and centrum ovale majus after prednisone were reduced to 15 mg/d. The new neurological deficits and multiple lesions after prednisone withdrawal might support this conclusion. This also suggested that the inhibitory potency of glucocorticoids for the recurrence of MS might be related to its measurement [14].

This patient with MS seemed to be only sensitive to glucocorticoids. MS recurred during the treatment of mycophenolate mofetil and teriflunomide, possibly due to progressive MS [15]. In addition, disease-modifying therapy (DMT) can reduce the risk of recurrence of relapsing-remitting MS by 36%–85%, although different drugs may have different effects. Some studies suggested that MS patients who relapsed repeatedly, before or shortly after DMT, might relapse uncontrollably despite continuous DMT [16–19]. Anti-NMDAR encephalitis and CNS demyelinating diseases can overlap, but the underlying mechanism is still unclear [20]. For the patient described in this study, the pathogenesis may be a common autoimmune response [20]. The occurrence of anti-NMDAR encephalitis may cause immunologic imbalance and induce MS [21]. In addition, NMDARs are partially expressed on oligodendrocytes that form myelin, and demyelination is evoked when anti-NMDAR encephalitis occurs [22]. Conversely, MS can also expose NMDAR antigens [21, 23].

## Conclusions

In conclusion, the mechanism of overlapping anti-NMDAR encephalitis and MS may be the coexistence of autoimmune reactions. The symptoms, MRIs, and immunological examinations of CSF or serum are often atypical. Thus, the positive antibody cannot be used as the only diagnostic basis. Furthermore, the overlap cannot be arbitrarily treated as the recurrence of a previous disease. Long-term follow-up, dynamic antibody monitoring, and MRI examination are crucial for these patients. For the patient described in this study, only glucocorticoids could control the recurrence. So, we speculate that this may be related to the short overlap time between the administration of glucocorticoids and DMT. Long-term use of the combination of low-dose effective glucocorticoids and DMT may be effective against the relapse of overlapping anti-NMDAR encephalitis and MS.

## Data Availability

The datasets are available from the corresponding author on reasonable request.
